# DESTINY of Immunotherapy in Patients With *HER2* Mutant Lung Cancer: Case Report

**DOI:** 10.1016/j.jtocrr.2025.100818

**Published:** 2025-02-28

**Authors:** Lorenza Landi, Francesca Fusco, Enrico Melis, Simonetta Buglioni, Paolo Visca, Gabriele Minuti, Federico Cappuzzo

**Affiliations:** a Clinical Trial Unit, Phase 1 and Precision Medicine, IRCCS Regina Elena National Cancer Institute, Rome, Italy; b Medical Oncology 2, IRCCS Regina Elena National Cancer Institute, Rome, Italy; c Thoracic Surgery Department, IRCCS Regina Elena National Cancer Institute, Rome, Italy; d Department of Pathology, IRCCS Regina Elena National Cancer Institute, Rome, Italy

**Keywords:** Lung cancer, HER2 mutation, Immunotherapy, Pathologic complete response, Case report

## Abstract

Immunotherapy has significantly transformed the landscape of non–oncogene-driven NSCLC. Multiple trials have reported that immunotherapy, either alone or in combination with chemotherapy, can induce pathologic complete responses—essentially tumor eradication—in non–oncogene-driven patients, at least in resectable stages. In contrast, tumors with specific molecular alterations, such as *EGFR* mutations or *ALK* rearrangements, are generally considered refractory to immune checkpoint inhibitors. Data on other lung cancer drivers, including *HER2* mutations, are scarce and limited to retrospective series, raising the question of whether immunotherapy should be avoided in all oncogene-addicted lung cancers. Here, we present the case of a patient with advanced *HER2* mutant NSCLC who achieved complete pathologic response to chemoimmunotherapy. Our observation suggests that not all driver mutations equally drive resistance to immunotherapy, and some molecular events, such as *HER2* mutations, need additional investigations.

## Introduction

Immunotherapy dramatically changed the natural history of non–oncogene-driven NSCLC, with a fraction of patients being long survivors.^1^ First-line treatment with programmed cell death protein-1 or programmed death-ligand 1 (PD-L1) inhibitors, as monotherapy or combined with chemotherapy, represents the standard treatment for patients with advanced NSCLC without target alterations. More recently, several trials reported that immunotherapy alone or combined with chemotherapy induced pathologic complete response (pCR) in a group of patients with non–oncogene-driven NSCLC, at least in resectable stages.^2^ Conversely, tumors harboring molecular alterations, such as *EGFR* mutations or *ALK* rearrangements, are generally considered refractory to immune checkpoint inhibitors. Among genetic alterations, *HER2* mutations are detected in approximately 2% to 3 % of patients with lung adenocarcinoma.^3^ For patients with *HER2* mutant NSCLC, data about immunotherapy efficacy are scarce and limited to retrospective series, raising the question of whether immunotherapy should be avoided in all oncogene-addicted lung cancers. Even if the effectiveness of targeting *HER2* in lung cancer has been known since 2006,^4^ the first agent gained FDA approval only in 2022. Trastuzumab deruxtecan is now considered the most effective agent in advanced *HER2* mutant NSCLC, leading to a response rate exceeding 50% in the DESTINY trial.^5^ Nevertheless, the complete remission rate is below 2%, suggesting that a targeted therapy is unlikely to eradicate a tumor.

Here, we present a case of a patient with advanced *HER2* mutant NSCLC who achieved pCR to chemoimmunotherapy.

## Case presentation

**Figure 1:**
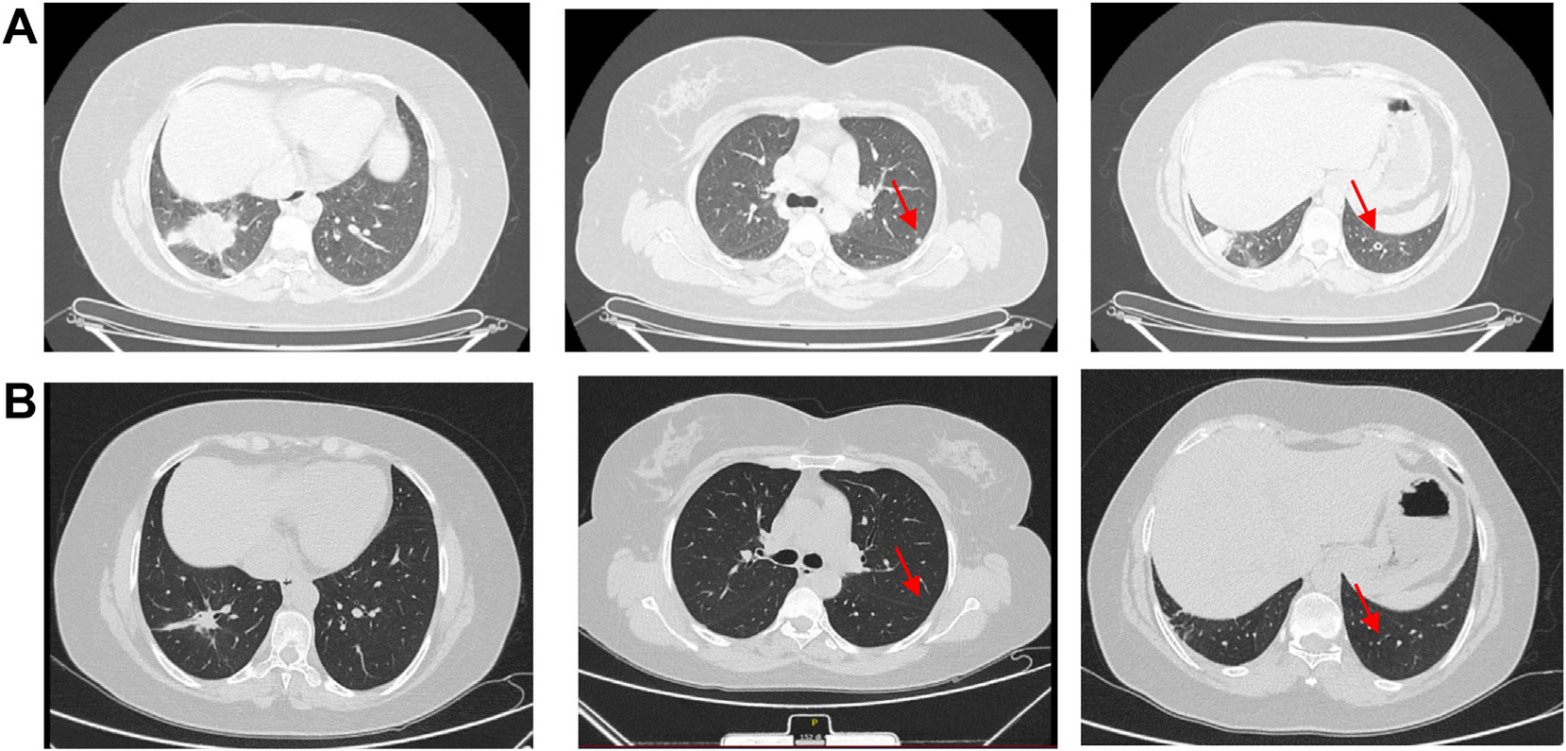
Response to chemoimmunotherapy in a patient with *HER2* mutant NSCLC. Adenocarcinoma of the right lung with metastasis in the mediastinal node and contralateral lung was diagnosed in August 2021. CT scan performed after four courses of cisplatin-pemetrexed-pembrolizumab reported a partial response in the primary lesion and a complete response of all secondary left pulmonary nodules. The response lasted for approximately 24 months *(A, B).* CT, computed tomography.

**Figure 2:**
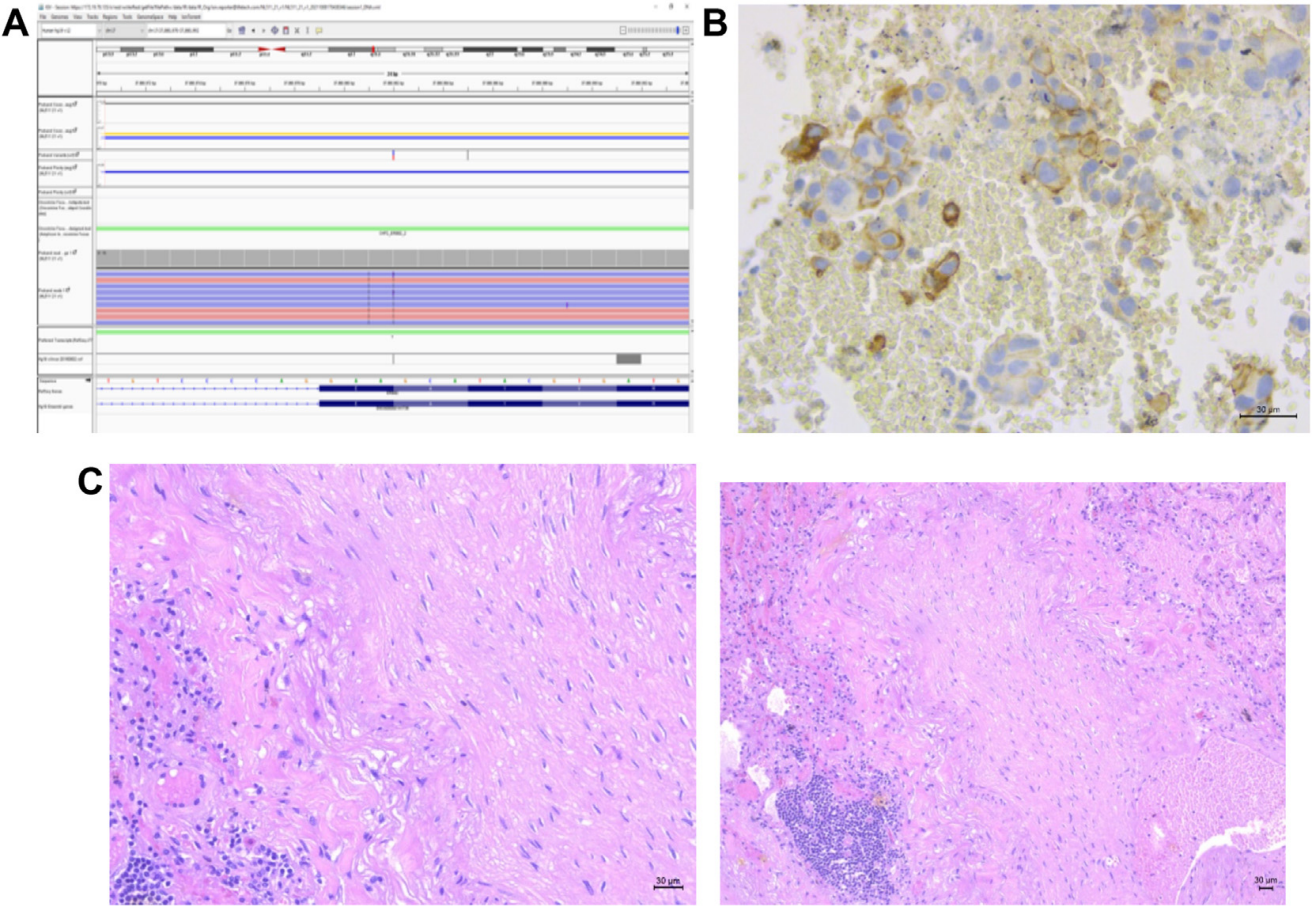
Molecular analysis of tumor sample and pathologic examination after surgery. Next-generation sequencing found the presence of the *HER2* exon 20 mutation p.(Y772_A775dup), whereas the PD-L1 TPS was 50% *(A, B).* Considering the depth and duration of response and the persistence of only a single lesion, as per the patient’s request, a right lower lung lobectomy with mediastinal lymphadenectomy was performed. Pathologic examination reported a pathologic complete response *(C).* PD-L1, programmed death-ligand 1; TPS, tumor proportion score.

**Figure 3:**
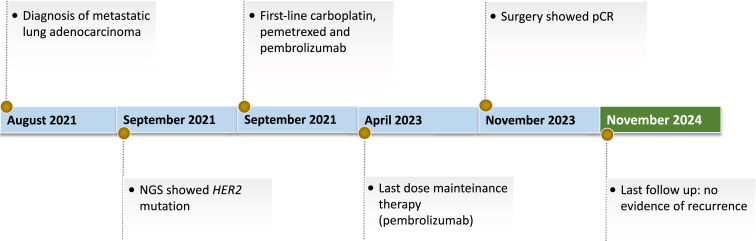
Timeline of the whole course of treatment. NGS, next-generation sequencing; pCR, pathologic complete response.

In September 2021, a 43-year-old female patient with metastatic lung adenocarcinoma was referred to our Institute (Fig. 1*A*). Controlateral lung was the only metastatic site. The PD-L1 tumor proportion score was 50%, and next-generation sequencing analysis (Oncomine Focus Assay Panel) reported an exon 20 insertion mutation in the *HER2* gene (Fig. 2*A* and *B*). A broader next generation sequencing panel (Oncomine Comprehensive Assay Plus Panel) was also performed, which confirmed exon 20 insertion mutation in the *HER2* gene p.(Y772_A775dup) and reported a mutation in *TP53* gene p.(R248W), microsatellite stability, low tumor mutational burden and low genomic instability status. Interestingly, the tumor cellularity percentage was 38% and the observed variant allele frequency was 34% for both *HER2* insertion mutation and *TP53* mutation. Even if mono-immunotherapy is the only approved option in PD-L1 overexpressing NSCLC and no anti-HER2 agent is available in Italy, the molecular portrait of this never-smoker patient led us to choose front-line chemoimmunotherapy. After four cycles, she obtained a partial response in her primary lesion with the disappearance of all secondary nodules in her left lung (Fig. 1*B*). She continued with maintenance therapy until April 2023, when mild renal toxicity occurred. At that point, as tumor reassessment remained unchanged with the only persistence of the primary lesion, the patient asked for surgical resection. Despite the multidisciplinary board voting against surgery, she was determined to remove the residual mass, and in November 2023, she underwent a right lower lobectomy with radical lymphadenectomy with evidence of pCR (Fig. 2*C*). After surgery, no additional therapy was offered. The whole course of treatment is summarized in Figure 3.

The patient provided written informed consent for tests, treatment, and publication.

## Discussion

For patients with NSCLC, treatment decisions are based on an accurate determination of stage and histologic diagnosis, including molecular profiling. The efficacy of targeted therapies in *HER2*-mutant lung cancer has been known since our first report in 2006. Nevertheless, the optimal treatment sequencing is still debated, and clinical trials are investigating the role of first-line *HER2*-inhibitors (e.g., trastuzumab deruxtecan, zongertinib) compared with chemoimmunotherapy. The other major challenge is the treatment of patients with brain metastases, which occur in nearly half of patients with *HER2*-positive NSCLC, and where *HER2*-targeted agents may play a role. Although it is known that not all oncogenic drivers respond better to first-line chemoimmunotherapy compared with chemotherapy alone, as shown in the LIBRETTO-431 trial where efficacy results in the overall intention-to-treat population were similar to those in the intention-to-treat pembrolizumab population, immunotherapy data are still lacking for *HER2*-positive individuals. Our case provides evidence that *HER2*-mutant lung cancer could benefit from chemoimmunotherapy treatment, although the oligometastatic stage, amenable to integrated treatments, and the high PD-L1 expression may have contributed to the exceptional response observed. Nevertheless, complete remission has been pathologically confirmed, demonstrating that immunotherapy could potentially eradicate the tumor, even in the presence of a driver and even in advanced disease.

## Conclusion

Our case is the first evidence that *HER2* mutant lung cancer could benefit from chemoimmunotherapy. The impressive and unexpected response observed reinforces the concept that not all molecular targets equally drive resistance to immunotherapy and that immune system modulation could represent an important strategy, at least in some selected cases.

## CRediT Authorship Contribution Statement

**Lorenza Landi:** Conceptualization, Data curation, Investigation, Writing - original draft.

**Francesca Fusco:** Conceptualization, Data curation, Writing - review & editing.

**Enrico Melis:** Visualization.

**Simonetta Buglioni:** Resources, Visualization.

**Paolo Visca:** Resources, Visualization.

**Gabriele Minuti:** Visualization.

**Federico Cappuzzo:** Conceptualization, Data curation, Investigation, Writing - review & editing.

## Disclosure

Dr. Landi reported fees for membership of an advisory board or lectures from Pfizer, Novartis, AstraZeneca, Roche, Takeda, Bristol-Myers Squibb, Merck Sharp & Dohme, Eli Lilly, Amgen, and Sanofi. Dr. Minuti reported fees for membership of an advisory board, lecture, or medical writer from Astra-Zeneca, Roche, Bristol-Myers Squibb, Merck Sharp & Dohme, Gilead, Novartis, Amgen, Sanofi, Daiichi Sankyo, and Johnson & Johnson. Dr. Cappuzzo reported fees for membership of an advisory board or lectures from Roche, AstraZeneca, Bristol-Myers Squibb, Pfizer, Takeda, Eli Lilly, Bayer, Amgen, Sanofi, Pharmamar, Novocure, Mirati, Galecto, OSE, ILLUMINA, Thermofisher, and Merck Sharp & Dohme. The remaining authors declare no conflict of interest.
